# Isothermal Crystallization and Rheology Properties of Isotactic Polypropylene/Bacterial Cellulose Composite

**DOI:** 10.3390/polym10111284

**Published:** 2018-11-18

**Authors:** Bo Wang, Fu-hua Lin, Xiang-yang Li, Zhong-wei Zhang, Xiao-rong Xue, Si-xiao Liu, Xu-ran Ji, Qian Yu, Zheng-qiu Yuan, Xin-de Chen, Jun Luo

**Affiliations:** 1 School of Chemistry and Biological Engineering, Taiyuan University of Science and Technology, Taiyuan 030024, China; 13546474299@163.com (B.W.); zzw18735374641@stu.tyust.edu.cn (Z.-w.Z.); Xuexiaorong22@stu.tyust.edu.cn (X.-r.X.); liusixiao@stu.tyust.edu.cn (S.-x.L.); jxr12345@stu.tyust.edu.cn (X.-r.J.); yq123@stu.tyust.edu.cn (Q.Y.); 2 Key Laboratory of Renewable Energy, Guangzhou Institute of Energy Conversion, Chinese Academy of Sciences, Guangzhou 510640, China; kelin0514@163.com (F.-h.L.); yuanzhengqiu@126.com (Z.-q.Y.); 3 Shanxi Provincial Institute of Chemical Industry, Shanxi 030021, China; 17835611229@163.com; 4 School of Chemistry and Chemical Engineering, Hunan University of Science and Technology, Xiang Tan 411201, China; 5 Guangzhou Fibre Product Testing and Research Institute, Guangzhou 510220, China

**Keywords:** Isotactic polypropylene, bacterial cellulose, rheology, isothermal crystallization

## Abstract

Bacterial cellulose (BC) is a new kind of cellulose with great potential in enhancing preparation of isotactic Polypropylene (iPP) composites, which have been found with excellent performance. However, the interface compatibility between BC and iPP is poor. In this study, iPP/BC composites were prepared by solution mixing. Esterification modified BC (CO) and Maleic anhydride grafted polypropylene (MAPP) added as a compatibilizer was both used to improve the interfacial compatibility of the iPP/BC composites. The rheology and isothermal crystallization behavior of the composites was tested and discussed. The result shows that the complex viscosity and storage modulus of the composite significantly increase in the rule iPP, iPP/BC2, iPP/CO2, and M-iPP/BC3, which indicates that the compatibility of the composite increases as this rule. According to the isothermal crystallization kinetics result, the crystal growth mode of iPP was not affected by the addition of BC and the interfacial compatibility. The spherulite growth rate of the iPP/BC composite increases with increasing crystallization temperature. Especially, the value decreases as the same rule with the complex viscosity and storage modulus of the composite at the same isothermal crystallization temperature. These results suggest that the interface compatibility of iPP/BC composites is greatly improved and the interface compatibility of the M-iPP/BC3 is better than the iPP/CO2.

## 1. Introduction

Isotactic propylene (iPP) is an important commercial plastic and has been widely used as matrix components in automobile, textile, packaging, construction, and other industrial uses [1,2]. The iPP has a lot of valuable properties such as low density, high strength, chemical resistance, wear-resisting, and low price [3]. Meanwhile, iPP has a variety of molding methods, including injection molding, extrusion molding, and blow molding [4]. However, the poor impact resistance of iPP limits its applications [5]. Aiming at this problem, many modification methods have been proposed. Among them, blending modification has advantages of convenient operation, good modification performance and economy [6,7]. Inorganic particles (Nano CaCO_3_, Nano SiO_2_, and Clay) [8,9,10], and fibres (Glass fibre, Polymer fibre and Nature fibre) [11,12,13] are widely used as fillers in blending modification of iPP to prepared iPP composites. Based on the cheap, renewable and biodegradable nature fibre, the iPP/ natural fibre composites have been extensively studied and used due to their good mechanical properties, renewable character, and lightness [14]. However, due to the complexity of natural fibre composition (cellulose, hemicellulose, and lignin), the uniformity of the composite cannot be guaranteed. Due to that, pure cellulose, used as filler to prepare iPP composites, has attracted more and more attention [15,16].

Bacterial cellulose (BC) is a new kind of cellulose with great potential in many fields, such as materials, food, medicine, etc. [17]. Compared with natural fiber, BC is made of superfine fiber network and has advantages of artificially controlled synthesis, high purity, high crystallinity, high degree of polymerization, good mechanical properties, and biodegradability [18,19], and it can be used to form complementary to the structure and properties of iPP, therefore overcomes the shortcomings of iPP, and greatly broadens the scope of iPP application.

Unfortunately, BC shows strong surface polarity because of the hydroxyl group in the molecular chain of BC, which leads the interface compatibility and the dispersion of BC and iPP are poor, the stress cannot be effectively transmitted between the interface, the properties of the composites are not satisfactory [20,21].

Currently, the commonly used methods for improving the interface quality mainly include reducing the surface polarity of BC (Esterification) and adding the compatibilizer (Maleic anhydride grafted polypropylene, MAPP) [22,23,24,25]. The mechanism of both the methods is to form covalent links with the hydroxyl groups present at the surface of the BC [26]. In our previous research, esterification modified BC (CO) and adding MAPP as compatibilizer were both used to improve interfacial compatibility of iPP and BC. The results indicate that the mechanical properties of iPP has been enhanced, and the effect of MAPP addition is better than that of esterification modified BC [27,28].

Besides, the rheology and crystallization behavior have a special place in polymer blends system, since it partly controls the compatibility and mechanical properties of the composite, and the filler may affect crystallization kinetics, degree of crystallinity, crystalline morphology, and crystal type of the composite [26,29]. The crystallization kinetics of the polymer composites is dived into non-isothermal crystallization and isothermal crystallization. Recently, our research group has reported the non-isothermal crystallization of the iPP/BC composites. Results show that the non-isothermal crystallization rates ascend in the order; the compatibility of iPP/BC composites and the cooling rate have great influence on the non-isothermal crystallization behavior of iPP/BC composites [30].

It is worth mentioning that the rheological behavior of the polymer composite is sensitive to the structure, dispersion, shape, surface modification, and particle size of the filler. However, the degree of dispersion of the filler, shape, and size of the polymer matrix and filler compatibility are closely related. Due to that, understanding the rheology is not just important to the optimization of processing behavior, and is also a potential method for direct evaluation of dispersion state of the composites in melt state. Therefore, rheology can be used as a tool to supplement traditional polymer composites characterization methods, such as SEM, TEM, and X-ray scattering. Study on rheology of polymer composites compatibility is that measurement is in the molten state, a series of different rheological parameters such as complex viscosity (η*), storage modulus (*G*′), and loss modulus (*G*″) can be used to study the overall structure response of composites on the linear and nonlinear deformation [29].

In this study, the iPP/BC composites were prepared by solution mixing. The rheology behaviors of iPP/BC composites were perfored by the rotational rheometer. The effect of compatibility of iPP/BC composites on isothermal crystallization kinetics, spherulite growth rate is also discussed.

## 2. Materials and Methods

### 2.1. Feedstock

iPP (S1003) was purchased from Sinopec Beijing Yanshan Company (Beijing, China) with melt flow index of 3.6 g/10 min. MAPP (CMG9801) was purchased from Nantong Sunny Polymer New Material Technology Co., Ltd (Nantong, China) with percent grafting at 0.8–1.2%, and melt flow index of 20–100 g/10 min (190 °C/2.16 kg). The BC film (Hainan Yida Food Industry Co., Ltd., Hainan, China) was flushed with deionized water to neutral, dried by freeze-drying machine, then milled into powder (20–200 nm). Pyridine and caprylyl chloride were purchased from Aladdin Industrial Corporation (Shanghai, China).

### 2.2. Preparation of CO

First, BC powder was dispersed in pyridine with stirring (*w*/*w*, 1:20) at 80 °C for 30 min. Caprylyl chloride (with the mass ratio of BC 30:1) was added dropwise into the system. After 7 h, the solid product of the system was washed with ethyl alcohol and deionized water to neutralize the product and dried in an air-oven for 12 h, and CO was obtained [30]. Under this condition, the substituting degree of CO was 2.32 [27].

### 2.3. Preparation of iPP/BC Composites

In this study, solution blending was used to prepare iPP/BC composites. The formula of iPP/BC composites is illustrated in Table 1. Each component was added into three flasks with 100 mL xylene at 130 °C and stirred for 6 h. Then, the solution was poured into the ice-methanol to precipitate the iPP/BC composites. After that, the iPP/BC composites were filtered by Brinell funnel. At last, the composites were dried at 80 °C for 12 h and the iPP/BC composites were obtained.

### 2.4. Rheological Properties Test

The melt rheological properties of the iPP/BC composites were determined in the oscillatory mode by a rotational rheometer (Kinexus ultra+, Malvern Instruments Ltd., Worcestershire, UK). The measurements were performed in the dynamic frequency scanning (100–0.1 Hz) using a strain of 2% at 200 °C. The strain was located within the linear viscoelastic range of the sample [31].

### 2.5. Differential Scanning Calorimeter (DSC)

The isothermal crystallization of the iPP/BC composites was performed on a DSC (Q2000, TA Instruments, Chicago, IL, USA). Samples were weighted 10 ± 1 mg, enclosed in Aluminum pans, firstly heated to 220 °C, maintained at 200 °C for 5 min to erase thermal history, then cooled to 130, 135, 140 °C (cooling rate = −80 °C /min), respectively. The isothermal crystallization was set as 400 min. The heat flows during both crystallization processes were recorded for data analysis.

### 2.6. Polarized Optical Microscope (POM)

The POM observations were carried out on a polarized optical microscope (BX51, Olympus Corporation, Tokyo, Japan) with a camera (COOLPIX4500, Nikon Corporation, Tokyo, Japan). Hot stage (LTS 350, Linkam Scientific Instruments, Tadworth, UK) was used to control the temperature of the sample. For preparation of the sample of POM test, the samples were pressed into sheeting (50 μm) by a heating pressing machine (140 °C, 12 MPa). 

## 3. Result and Discussion 

### 3.1. Rheological Properties of the iPP/BC Composites

Generally, in the case of homopolymers, the flow behavior depends on the flow geometry and processing conditions. In the case of polymer blends, the flow behavior becomes more complex and is influenced by additional factors such as the miscibility of the system, the morphology, the interfacial adhesion, and the interfacial thickness. As we know, MAPP is forced to locate at the interface between iPP and BC phase. The polar functional groups in MAPP interact with the polar functional groups in BC, and the iPP backbones can be compatible with the iPP in the blends. This leads to the decrease of interfacial tension and the improvement of interfacial adhesion. As a result, the minor phase (BC) disperses in the major phase (iPP) matrix. This causes an increasing trend of chemical interaction between the distinct phases. Due to this, the compatibility of iPP and BC may be reflected in their rheological properties.

**Figure 1:**
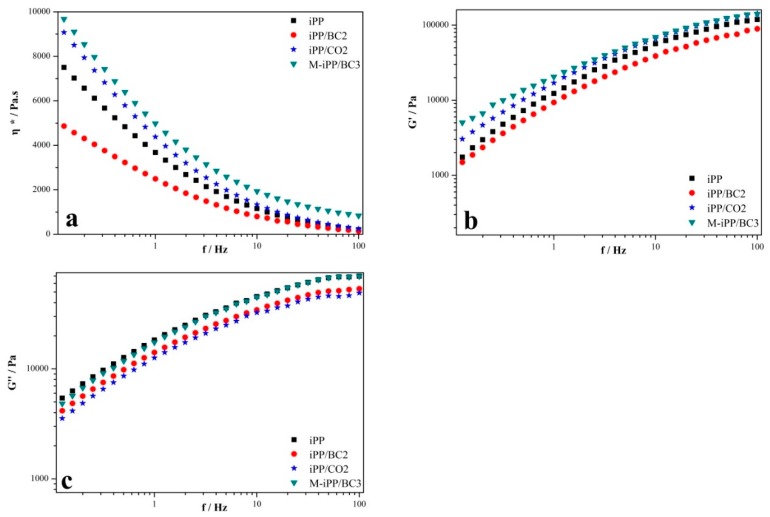
The rheological properties of the iPP/BC composite. ((**a**) complex viscosity, (**b**) storage modulus, and (**c**) loss modulus)).

The rheological properties of iPP/BC composites were characterized in order to study the compatibility of iPP and BC. The complex viscosity of the iPP/BC composites is shown in Figure 1. It can be found that the complex viscosity of the composites reduce sharply in the range of high frequencies. This phenomenon reveals that the composites present evidence of shear-thinning behavior with increasing frequencies [29]. It can be found that the complex viscosity and storage modulus of the composites increase in the order of iPP/BC2, iPP, iPP/CO2, and M-iPP/BC3. Moreover, the addition of MAPP to iPP evidently increases the complex viscosity, especially at low frequencies, and this can be attributed to the formation of a two-phase structure. The existence of interfacial interactions between iPP and BC leads to the increase in the viscosity of the blend [32]. Usually, the energy storage mechanisms at the interphase of polymer and filler reflect the elastic properties of the composites. The relaxation of the BC is often longer than the relaxation of the iPP, which means the storage modulus of iPP/BC is lower than pure iPP [33].

In addition, the loss modulus of the composites shows no obvious regularity, which reveals that the sensitivity of loss modulus in the characterization of the compatibility is relatively low. But the M-iPP/BC3 shows higher loss modulus values than the iPP/BC2 and iPP/CO2. This is because the compatibilizer MAPP and curing can further enhance the values over the high frequencies. In the terminal zone, in which only the longest relaxation time contributes to the viscoelastic behavior, loss modulus of linear polymers follow the well-known frequency dependence.

### 3.2. Isothermal Crystallization Kinetics of the iPP/BC Composites

**Figure 2:**
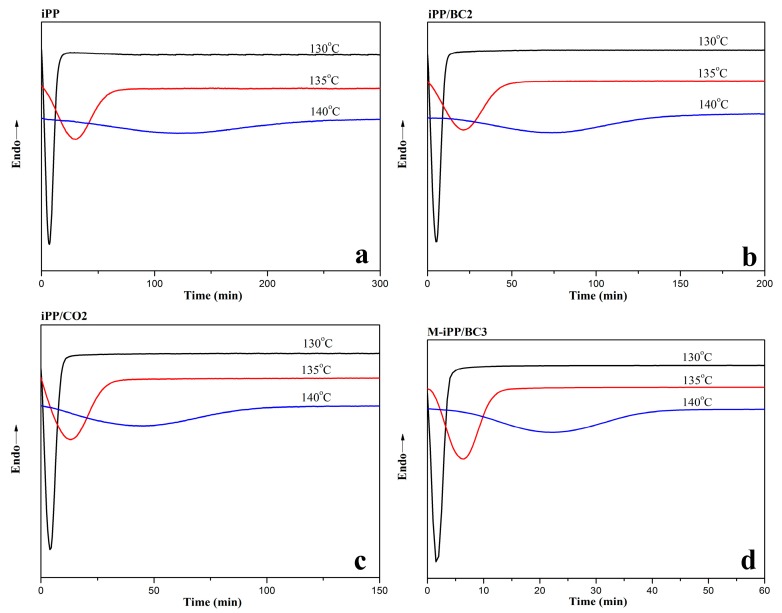
Isothermal crystallization curves of iPP/BC composites at different temperatures. (**a**) iPP (**b**) iPP/BC2 (**c**) iPP/CO2 (**d**) M-iPP/BC3.

Generally, the isothermal crystallization kinetics of polymer and polymer blends is characterized by the DSC date at a different temperature. To analyze the effects of interface compatibility and isothermal temperature on the crystallization kinetics for the iPP/BC composites, the isothermal crystallization temperature has been set as 130, 135, and 140 °C. The DSC crystallization curves for iPP/BC composite are presented in Figure 2. It is observed that the peak width of crystallization peak decrease in the order of iPP, iPP/BC2, iPP/CO2, and M-iPP/BC3, which indicates that the crystallization rate from slow to fast follows this rule. Besides, the crystallization peak becomes broader with the isothermal crystallization temperature increases, which implies that the crystallization rate decreases and the time needed for the completion of crystallization increases [34]. The phenomenon proves that the BC can greatly enhance the crystallization rate of iPP as a heterogeneous nucleation point. This is further evidence to demonstrate the changes in the compatibility of the composites.

The classical Avrami equitation, which is given in Equation (1), is used to study the isothermal crystallization kinetics of iPP/BC composites [35,36].

(1) $${1-X}_{v}\left(t\right)=\exp\left({1-kt}^{n}\right)$$

where *X_v_*(*t*) is the relative degree of crystallinity of the sample at *t* time, *k* is the crystallization rate parameters which involving both nucleation and growth rate, and *n* is the Avrami exponent depending on nucleation type and the crystal growth geometry. 

**Figure 3:**
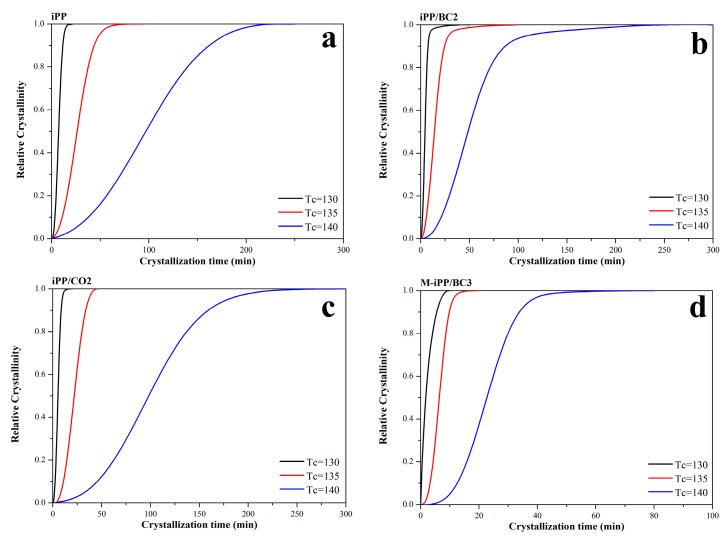
Relative degree of crystallinity with time of iPP/BC composites. (**a**) iPP (**b**) iPP/BC2 (**c**) iPP/CO2 (**d**) M-iPP/BC3

As shown in Figure 3, the DSC results provide relative degree of crystallinity, *Xv*, which are calculated by the ratio of exothermic peak area at the crystallization time, *t* and the total area under the exothermic peak, as shown in Equation (2):

(2) $$X_{v}\left(t\right)=\int_{0}^{t}\left(\frac{dH}{dt}\right)dt\int_{t_{0}}^{t_{\infty }}\left(\frac{dH}{dt}\right)dt$$

where *dH/dt* is the rate of heat evolution, *t*_0_ and *t_∞_* are crystallization start and end times, respectively.

For convenience of calculation, Equation (1) is usually rewritten in a double logarithmic form as follows:

(3) $${\log[-\ln(1-X}_{v}\left(t\right))]=n\log t+\log k$$

According to Equation (3), when the log[−ln(1−*X_v_*(*t*))] is plotted against log*t*, the Avrami exponent, *n*, and crystallization rate constant, *k* can be directly obtained as the intercept and slope of the linear plot of log[−ln(1−*X_v_*(*t*))] versus log*t*, respectively.

The half-time of crystallization (*t*_1/2_) can be calculated from Equation (1) if *Xv*(*t*) = 0.5, which is Equation (4), as shown:

(4) $$t_{1/2\;}{=(\ln2/k)}^{1/n}$$

**Figure 4:**
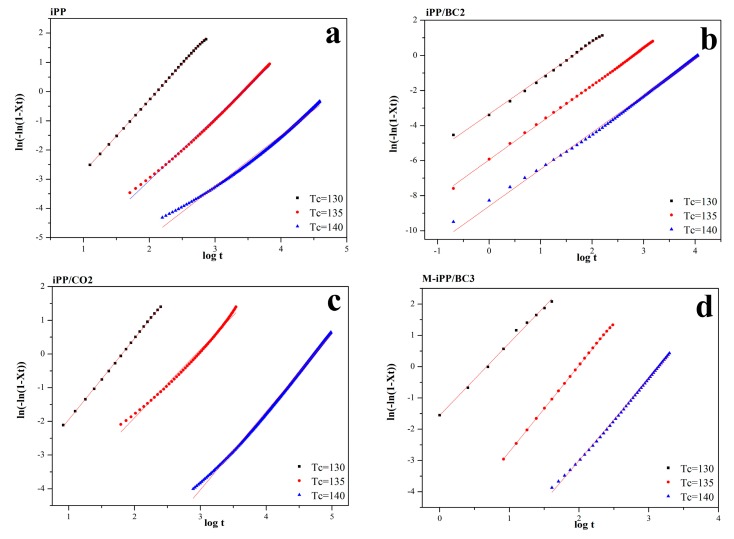
Isothermal crystallization kinetics curves of the iPP/BC composites. (**a**) iPP (**b**) iPP/BC2 (**c**) iPP/CO2 (**d**) M-iPP/BC3.

The isothermal crystallization kinetics curves and the isothermal crystallization kinetic parameters for iPP/BC composites obtained at various temperatures are illustrated in Figure 4 and Table 2. 

The results indicate that the isothermal crystallization kinetics curves of the iPP/BC composites are basically linear, which shows that the Avrami equation can describe the isothermal crystallization mechanism of iPP/BC composites well. First of all, the overall crystallization rate (*t*_1/2_) of the iPP/BC composites significantly decreases with increasing crystallization temperature. However, the value slightly decreases in the order of iPP/BC2, iPP, iPP/CO2, and M-iPP/BC3. Moreover, the value *t*_1/2_ is close to *t*^’^_1/2_. These results reveal that the iPP/BC composites have higher crystallization ability at a lower temperature, and with the compatibility of iPP/BC composites improves, the overall crystallization rate increases. Secondly, the *n* value of all the composites is approximately two, and the *n* value of the composites increases with the increase of crystallization temperature. This phenomenon indicates that the crystal growth mode of iPP is not subject to the addition of BC, and the increase of crystallization temperature can make the crystal of iPP much perfect. Importantly, with the improvement of the interface compatibility of iPP and BC, the crystal perfection of iPP increases. Furthermore, the effect of added MAPP is better than esterification modified BC. Interestingly, the change of crystallization rate constant (*k*) is exactly the same as *t*_1/2_ and *n*, which is another evidence of improvement of the interface compatibility of iPP and BC with the modification. 

### 3.3. Spherulite Growth Rate of the iPP/BC Composites

**Figure 5:**
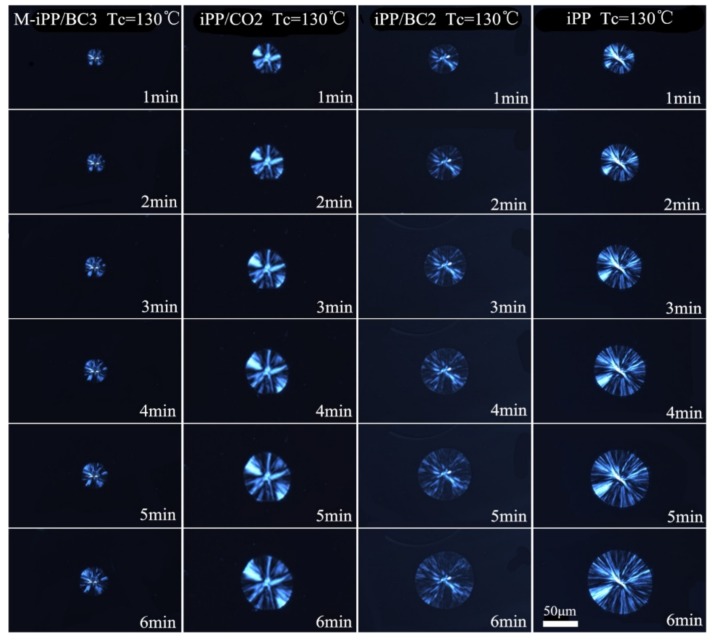
The POM images of the iPP/BC composites at 130 °C.

**Figure 6:**
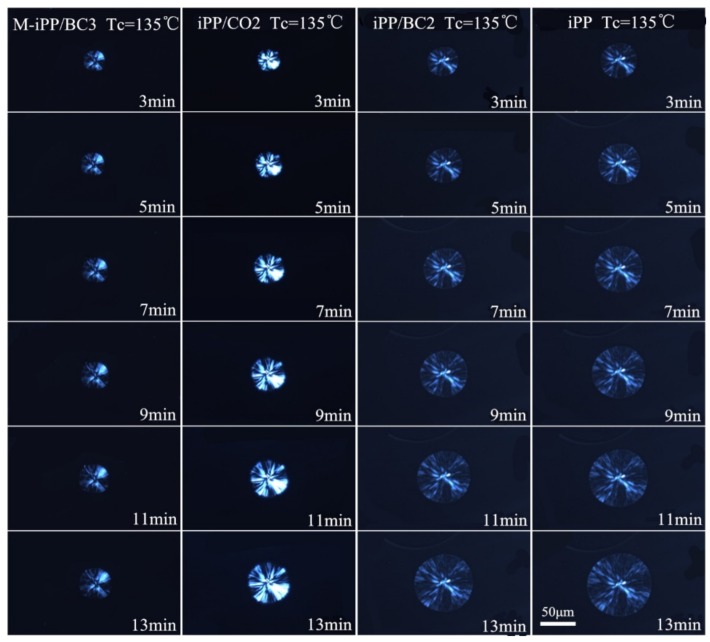
The POM images of the iPP/BC composites at 135 °C.

**Figure 7:**
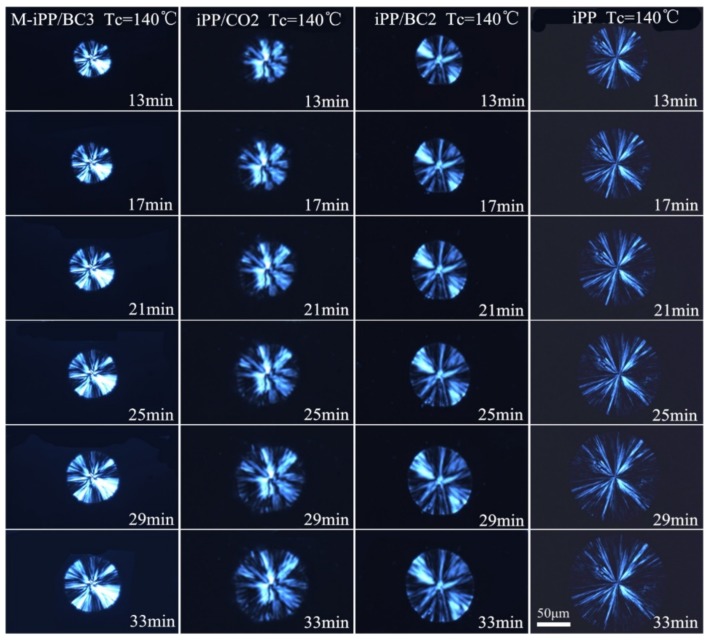
The POM images of the iPP/BC composites at 140 °C.

It is well known that the primary nucleation and linear crystal growth rates are both involving the overall crystallization rate by the Avrami equation. In order to investigate the effects of the crystallization temperature and the interface compatibility on the primary nucleation, the POM was used to analyze the isothermal crystallization process, as shown in Figure 5, Figure 6 and Figure 7. It can be found from the figures that with the crystallization temperature increases, the spherulite size increases, and with the improvement of interface compatibility of iPP and BC, the spherulite size decreases. 

**Figure 8:**
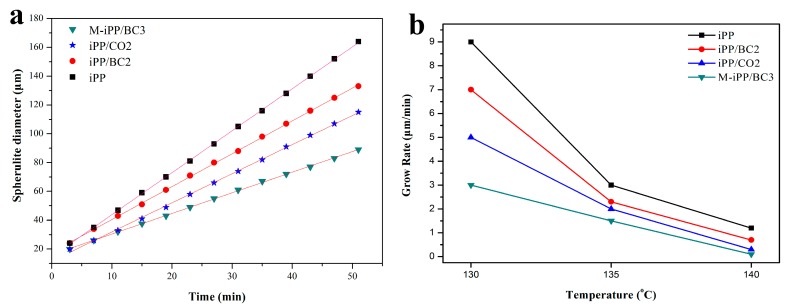
Effects of interface compatibility on (**a**) the changes of spherulite diameter at 135 °C and (**b**) the spherulite growth rate of the iPP/BC composites.

The related studies show that the growth rate of spherulites in most polymer blends is only related to the crystallization temperature, and some researchers believe that the growth rate of spherulites is nonlinear in non-isothermal condition [37,38]. In this paper, the POM images at 135 °C were taken as an example, the spherulite radial growth rate of the iPP/BC composites was calculated (Figure 8a). It can be found from Figure 8a that the diameter of spherulites presents a linear growth with the extension of crystallization time and the slope is the spherulite growth rate of iPP at this temperature. The spherulite growth rate at different isothermal crystallization temperatures can be obtained by the calculation of POM images at different temperatures (Figure 8b). It can be observed in Figure 8b that, with the decrease of crystallization temperature, the spherulite growth rate of the sample increase rapidly. This is because the crystallization rate of growth depends on the chains in the crystal diffusion and structured deposits. With the decrease of crystallization temperature, the melt viscosity increases, reducing the chain segment mobility, crystal diffusion rate of the chains decreases. At the same time, with the regular degree of isothermal crystallization temperature related accumulation rate increases. While the melt viscosity and degree of isothermal crystallization temperature are the influencing factors of spherulite growth rate of the polymer blends, the crystallization temperature of iPP is close to melting point, which is much higher than that of glass transition temperature. Therefore, the influence of crystallization temperature reduction on the degree of isothermal crystallization temperature is much higher than that on melt viscosity, which causes the spherulite growth rate to increase rapidly with the decrease of crystallization temperature in the experimental temperature range [39,40].

Besides, the diffusion and regular accumulation rate of the polymer chain segments have a great influence on the spherulite growth rate [41]. It can be found in Figure 8b, the spherulite growth rate of the iPP/BC composites decreases as the rule: iPP, iPP/BC2, iPP/CO2, and M-iPP/BC3 at the same isothermal crystallization temperature. This is because the regular accumulation rate of the iPP and BC is identical at the same isothermal crystallization temperature, and the effect of BC on the spherulite growth rate is mainly reflected in the diffusion velocity of the crystalline segments in the iPP melt. When the iPP crystal grows, BC hinders the diffusion of iPP chains toward the crystal growth direction, which leads the spherulite growth rate of iPP/BC composites to slower than that of pure iPP. From another point of view, the composites with BC will not produce large spherulites. In this case, the composites with excellent mechanical properties can be expected. In addition, this is evidence of the change in compatibility of the iPP/BC composites after the different modification.

## 4. Conclusions

In this work, iPP/BC composites were prepared by solution mixing. Esterification modified BC (CO) and MAPP, added as compatibilizer, were both used to improve the interfacial compatibility of the iPP/BC composites. The rheology behavior of the composites shows that the complex viscosity and storage modulus of the composite significantly increase in the rule iPP, iPP/BC2, iPP/CO2, and M-iPP/BC3, indicating that the compatibility of the composite increases as this rule. The DSC results show that the BC can greatly enhance the crystallization rate of iPP as heterogeneous nucleation point. The isothermal crystallization kinetics result shows that the crystal growth mode of iPP is not affected by the addition of BC or the interfacial compatibility. At last, with the decrease of crystallization temperature, the spherulite growth rate of the iPP/BC composites increases rapidly, and the spherulite growth rate of the iPP/BC composites decreases as the rule: iPP, iPP/BC2, iPP/CO2, and M-iPP/BC3 at the same isothermal crystallization temperature. These results indicate that the interface compatibility of iPP/BC composites is greatly improved and the interface compatibility of the M-iPP/BC3 is better than the iPP/CO2.

## Figures and Tables

**Table 1:** Formula of the iPP/BC composites.

Sample	iPP (g)	BC (g)	CO (g)	MAPP (g)
iPP	10	–	–	–
iPP/BC2	10	0.2	–	–
iPP/CO2	10	–	0.2	–
M-iPP/BC3	10	0.3	–	0.7

**Table 2:** The isothermal crystallization kinetic parameters of the iPP/BC composites.

Sample	*T*c (^o^C)	*n*	log*k*	*t*_1/2_ (min)	*t*_1/2_^’^ (min)
iPP	130	1.7	−5.23	7.35	7.0
	135	2.2	−7.34	26.33	25.09
	140	2.4	−8.48	97.52	87.52
iPP/BC2	130	2.0	−3.34	4.62	4.3
	135	2.1	−5.95	21.67	20.03
	140	2.2	−8.60	88.56	76.01
iPP/CO2	130	2.1	−4.36	4.18	4.0
	135	2.3	−5.94	14.34	13.21
	140	2.4	−11.01	48.87	40.28
M-iPP/BC3	130	2.2	−8.22	1.67	1.26
	135	2.3	−9.47	6.43	6.04
	140	2.4	−11.55	22.85	19.78
